# Arsenic is associated with reduced effect of folic acid in myelomeningocele prevention: a case control study in Bangladesh

**DOI:** 10.1186/s12940-015-0020-0

**Published:** 2015-04-10

**Authors:** Maitreyi Mazumdar, Md Omar Sharif Ibne Hasan, Rezina Hamid, Linda Valeri, Ligi Paul, Jacob Selhub, Ema G Rodrigues, Fareesa Silva, Selim Mia, Md Golam Mostofa, Quazi Quamruzzaman, Mahmuder Rahman, David C Christiani

**Affiliations:** Department of Neurology, Boston Children’s Hospital, 300 Longwood Avenue, Boston, MA USA; Department of Environmental Health, Harvard School of Public Health, 665 Huntington Avenue, Boston, MA USA; Dhaka Community Hospital, 190/1 Baro Moghbazar, Wireless Railgate, Dhaka, 1217 Bangladesh; Bangladesh Medical College, 14/A Dhanmondi, Dhaka, 1209 Bangladesh; Department of Biostatistics, Harvard School of Public Health, 655 Huntington Avenue, Boston, MA USA; Jean Mayer USDA Human Nutrition Research Center on Aging, Tufts University, 711 Washington Street, Boston, MA USA

**Keywords:** Arsenic, Neural tube defect, Myelomeningocele, Birth defect, Folate deficiency

## Abstract

**Background:**

Arsenic induces neural tube defects in several animal models, but its potential to cause neural tube defects in humans is unknown. Our objective was to investigate the associations between maternal arsenic exposure, periconceptional folic acid supplementation, and risk of posterior neural tube defect (myelomeningocele) among a highly exposed population in rural Bangladesh.

**Methods:**

We performed a case–control study that recruited physician-confirmed cases from community health clinics served by Dhaka Community Hospital in Bangladesh, as well as local health facilities that treat children with myelomeningocele. Controls were selected from pregnancy registries in the same areas. Maternal arsenic exposure was estimated from drinking water samples taken from wells used during the first trimester of pregnancy. Periconceptional folic acid use was ascertained by self-report, and maternal folate status was further assessed by plasma folate levels measured at the time of the study visit.

**Results:**

Fifty-seven cases of myelomeningocele were identified along with 55 controls. A significant interaction was observed between drinking water inorganic arsenic and periconceptional folic acid use. As drinking water inorganic arsenic concentrations increased from 1 to 25 μg/L, the estimated protective effect of folic acid use declined (OR 0.22 to 1.03), and was not protective at higher concentrations of arsenic. No main effect of arsenic exposure on myelomeningocele risk was identified.

**Conclusions:**

Our study found a significant interaction between drinking water inorganic arsenic concentration from wells used during the first trimester of pregnancy and reported intake of periconceptional folic acid supplements. Results suggest that environmental arsenic exposure reduces the effectiveness of folic acid supplementation in preventing myelomeningocele.

## Background

Neural tube defects are debilitating birth defects characterized by high rates of mortality and lifelong disabilities in surviving children. Neural tube defects occur when the neural plate fails to fold in the first 3 to 4 weeks of gestation, causing death or permanent damage to the spinal cord [1]. Neural tube defects are among the most common birth defects worldwide, and their prevalence varies from 5.3 per 10,000 live births in the United States [2] to more than 10 per 1,000 pregnancies in certain areas of China [3]. This variance likely reflects differing contributions from risk factors such as prevalence of obesity and diabetes, nutritional status, usage of folic acid supplementation and/or folic acid fortification of the food supply, genetic susceptibility, and the presence of environmental toxicants. Although studies have identified numerous risk factors, these account for less than half of all neural tube defects, suggesting that additional genetic and environmental risk factors remain to be identified [4].

Of particular concern is environmental exposure to arsenic, which induces neural tube defects (exencephaly, spina bifida occulta) in several animal models, including mouse [5], rat [6], hamster [7], and chick [8]. Animal studies have demonstrated that arsenic crosses the placenta and preferentially accumulates in the neuroepithelium of developing hamster, mouse, and monkey embryos [9]. The potential mechanisms of arsenic-induced neural tube defects include direct toxicity from reactive oxygen species to the developing neural plate [8], and disruption of maternal glucose metabolism [10]. In addition, folate deficiency may exacerbate arsenic toxicity. Arsenic metabolism is catalyzed by enzymes that require S-adenosylmethionine (SAM) as the methyl donor. SAM is eventually regenerated via a process that requires 5-methyltetrahydrofolate as a cofactor. Thus, arsenic metabolism is dependent on the folate supply. The associations between folate and arsenic toxicity have been intensively studied in recent years. In mouse models, mice with specific defects in folate transport had higher rates of neural tube defects after *in utero* arsenic exposure than wild-type mice similarly exposed [5,11]. Understanding the associations between arsenic and folate is particularly important for public health practitioners as folic acid supplementation and fortification is the primary strategy for neural tube defect prevention. In addition to preventing neural tube defects, folic acid is recognized to reduce the risk of other congenital anomalies, with the strongest evidence supporting folic acid’s role in reduction of cardiac malformations [12].

Exposure to arsenic through drinking water sources from groundwater is a global public health emergency that is particularly devastating in Bangladesh. According to survey data from 2000 to 2010, an estimated 35 to 77 million people in the country have been chronically exposed to arsenic in their drinking water in what has been described as the largest mass poisoning in history [13]. The most recent survey of wells (2010) reported that 42% of the water samples tested in Bangladesh had inorganic arsenic levels exceeding the World Health Organization drinking water guideline of 10 μg/L, and 27% had levels that exceeded the Bangladesh guideline of 50 μg/L. Many areas the inorganic arsenic concentrations in drinking water exceeded 1000 μg/L [14].

This study aimed to identify whether environmental arsenic exposure is associated with higher risk of neural tube defects, and whether environmental arsenic exposure modifies the protective effect of folic acid supplementation. No data are available on the incidence and prevalence of neural tube defects in Bangladesh due to the country’s lack of a systematic surveillance system for birth defects, although these malformations are fairly common in clinical practices [15]. We therefore conducted a case–control study that established a new case ascertainment system. We administered a protocol that measured inorganic arsenic concentrations in drinking water from sources used during the first trimester of pregnancy.

## Methods

### Study population

This case–control study was conducted between April and November 2013 in the communities served by Dhaka Community Hospital (DCH) in Bangladesh. The unstable political situation in Bangladesh in November 2013 required the termination of the study after enrolling 57 of the planned 60 cases and 55 of the planned 60 controls. DCH operates community health centers throughout rural areas of Bangladesh, many of which are located within regions that are high priorities for arsenic remediation projects by the Bangladeshi government and by nongovernmental organizations. The case ascertainment system we established included regular communication between DCH and district and union health officials, health centers, and midwives in the Pabna, Barisal, Rajshahi, Munshiganj, and Chittagong Districts, as well as referral from local hospitals (Dhaka Community Hospital, Bangladesh Medical College, Dhaka Shishu Hospital, and Bangabandhu Sheikh Mujib Medical University). Eligible cases were infants and children under age 1 year with myelomeningocele.Infants affected by anterior neural tube defects (e.g. anencephaly) were not included, as they were not expected to be reliably identified using our strategy. Most births in these areas occur at home, and the birth of infants who die soon after delivery, as is expected for infants with anencephaly, is often not reported.

To confirm case diagnoses, a pediatrician with expertise in classifying neural tube defects (O.S.I.) performed a physical examination or, if the subject was deceased, reviewed photographs. Pregnancy registries from DCH-affiliated health centers that were located in the same areas as the cases served as the source of controls. Controls were matched (1:1) by sex and age using the following method: potential controls were separated into groups corresponding to sex and birth quarter, and placement on the list of potential controls was assigned by random digit assignment. Once a case was enrolled, potential controls were approached in order of assignment on the list. Participation was 98% among potential cases and 83% among potential controls. Reasons for refusal to participate were similar between cases and controls, including the lack of desire to participate in any studies and the fear that giving blood will cause sickness. Our *a priori* power estimate was that our pilot study sample size would provide 82% power if the true odds ratio for myelomeningocele in infants exposed to high levels of arsenic relative to low was 3.5. For this estimate, we defined high-level arsenic exposure as levels within the top quartile of observed; we assumed a correlation coefficient between cases and controls of 0.2 and a Type I error of 0.05.

Informed consent was provided by parents before enrollment. The Human Research Committees at Boston Children’s Hospital (BCH) and at DCH approved this study.

### Questionnaires and medical history

Trained interviewers asked parents about their medical histories, including the use of medications during pregnancy, family history, water consumption, and reproductive history. Periconceptional folic acid supplementation was defined as reporting any intake of a folic acid-containing supplement before within the two months prior to awareness of the pregnancy. Nutritional intake of mothers was assessed using a food frequency questionnaire previously validated in rural Bangladeshi populations [16]. Children underwent a physical examination including evaluation for level of neural tube defect and presence of other congenital abnormalities.

### Arsenic exposure from drinking water

At the time of enrollment, a water sample was collected from the tube well each mother identified as her primary source of drinking water at the time when she became aware of her pregnancy. Water samples were collected in 50 ml polypropylene tubes (BD Falcon, BD Bioscience, Bedford, MA), preserved with Reagent Grade nitric acid (Merck, Germany) to a pH < 2, and stored at room temperature. Arsenic analysis was conducted by using inductively coupled plasma mass-spectrometry (ICP-MS) following US EPA method 200.8 (Spectrum Analytical, Inc., Agawam, MA USA). For quality control, instrument performance was validated by a spiked laboratory control sample (ICP, Analytical Mixture 12 Solution A, High-Purity Standards, Charleston, SC USA) with percent recoveries ranging from 98% to 107%. Of the 112 samples included in this analysis, 45 (41.2%) had an inorganic arsenic concentration below the 0.15 μg/L limit of detection (LOD). These samples were reassigned half the value of the LOD for statistical analyses. Families found to have drinking water inorganic arsenic concentrations ≥ 50 μg/L (Bangladesh standard) were advised to seek alternative drinking water.

### Blood collection and processing

Maternal blood samples were collected via venipuncture into two EDTA tubes and transported in an insulated container to Dhaka within one day of collection, after which they were stored at −20°C. Blood was centrifuged and plasma collected into microcentrifuge tubes and stored at −20°C. Blood/plasma samples were shipped to HSPH on dry ice.

### Folate analysis

Folate analyses were performed at the Vitamin Metabolism Laboratory at the Jean Mayer USDA Human Nutrition Research Center at Tufts University. We measured total folate of the plasma samples by microbial assay with the use of *Lactobacillus casei *[17]. We serially diluted 5 μL of each plasma sample and plated the samples in triplicate onto a 96-microtiter well plate with 150 μL of *L. casei* growth medium. We incubated the plates overnight in a 37°C humid incubator and measured the absorbance, which indicated microbial growth, with the use of a 96-well plate reader (PowerWave HT; BioTek Instruments Inc, Winooski, VT USA) at 595 nm. To test if any arsenic in plasma affected the microbial assay, we spiked 3 random samples with 5 ng/mL folic acid, and there were no inhibitory components detected in the plasma. The coefficients of variation for the assay using one plasma sample with high folate concentration and one sample with low folate concentration were 6.78% and 4.73%, respectively.

### Statistical analysis

Data were analyzed using SAS (version 9.3; SAS Institute, Inc. Cary, NC USA). We used unconditional logistic regression to calculate crude and adjusted odds ratios (ORs) as well as 95% confidence intervals (CI). We did not use conditional models because of the uneven numbers of cases and controls, but instead forced the matching variables of child age and sex into all models, as strategy suggested by Rothman and Greenland [18,19]. Data exploration suggested that the log-odds of case identification varied exponentially with levels of inorganic arsenic in well water. Consequently, log-transformed inorganic arsenic concentration was treated as a continuous variable in regression models. Potential confounding factors for the main effect of water arsenic included smoking status of each parent, medication use, betel nut use, birth order, folic acid use, family history of congenital defect, use of pesticides during pregnancy, place of birth (home, birth center, or hospital), and report of undergoing an ultrasound during pregnancy – the latter two variables were considered additional measures of socioeconomic status.

We used inverse probability weighting (IPW) to correct locations that presented very different exposure profiles of our control populations, a method recently reviewed succinctly by Seaman and White [20]. A large percentage of our controls were drawn from Birhampur Community Clinic (BCC) and Sirajdikhan Community Clinic (SCC). BCC and SCC currently participate in other research activities conducted by our group; therefore, the population in those sites had a higher probability of selection as a control. Using IPW, each individual in the study population was given a sampling weight that was the inverse of the probability of selection, in this case the inverse of the proportion of controls from the site from which the control was recruited. We investigated effect modification with reported prenatal folic acid supplementation by using interaction terms in our weighted logistic regression models.

## Results

The 57 cases of neural tube defects included 49 cases of lumbar and 8 cases of cervical myelomeningocele. None of the mothers reported alcohol consumption, smoking, betel nut use, or use of medications other than periconceptional folic acid supplements (all mothers denied use of anticonvulsant drugs) during pregnancy. None of the mothers reported a diagnosis of diabetes, including gestational diabetes. Overall, 39.4% of the study population reported periconceptional folic acid use, which is similar to other studies in Bangladesh [21]. As shown in Table 1, mothers of controls were younger than mothers of cases (23.1 years versus 25.2 years, p = 0.02). Mothers of controls were more likely to report periconceptional folic acid use than mothers of cases (71% versus 49%, p = 0.02). Plasma folate concentrations were higher at the time of study visit among women who reported periconceptional folic acid use (Mean 12.7 nmol/L vs. 7.4 nmol/L, p = 0.02). Plasma folate concentrations were not significantly different between mothers of controls and mothers of cases (Mean 10.6 nmol/L vs. 10.5 nmol/L, p = 0.97).

**Table 1:** **Characteristics of cases of myelomeningocele and controls in Bangladesh (mean ± SD, except where noted)**

**Characteristic**	**Controls (n = 55)**	**Cases (n = 57)**	***p*** **Value**
Age (months)	8.0 ± 4.9	5.9 ± 5.3	0.38
Male [n (%)]	32 (58)	34 (60)	0.87
Mother’s age at delivery (years)	23.1 ± 4.1	25.2 ± 5.4	0.02
Father’s age at delivery (years)	31.5 ± 5.4	32.5 ± 6.8	0.38
Reported prenatal folic acid use [n(%)]	39 (71)	28 (49)	0.02
First born	25 (46)	23 (40)	0.56
Cesarian section [n(%)]	24 (44)	23 (40)	0.72
Birth site			0.15
Hospital	23 (22)	14 (25)	
Birth center/clinic	11 (20)	15 (26)	
Home	24 (44)	28 (49)	
Reported any ultrasound during pregnancy [n(%)]	50 (91)	48 (92)	0.19
Plasma folate concentration (nmol/L)	10.6 ± 14.7	10.5 ± 13.8	0.97
Level of neural tube defect			
Lumbar [n (%)]		49 (86)	
Cervical [n (%)]		8 (14)	

Differences in median drinking water arsenic concentration were observed. Controls had higher drinking water inorganic arsenic concentrations, though this appeared to be due to the contribution of controls from one site only, Birhampur Community Clinic (BCC) in Pabna (n = 22). Data exploration showed that controls from sites aside from BCC had lower median water arsenic concentrations than cases (Table 2).

**Table 2:** **Distribution of water inorganic arsenic concentrations**

	**Controls**	**Controls excluding BCC** ^**1**^	**Cases**
First trimester drinking water (μg/L)			
n	55	33	54
Mean ± SD	49.4 ± 103.6	38.3 ± 101.6	19.8 ± 54.6
Median	6.9	<LOD^2^	0.7
25th percentile	<LOD	<LOD	<LOD
75th percentile	54.9	8.5	12.7
Lowest	<LOD	<LOD	<LOD
Highest	506.0	450.0	357.0

^1^BCC = Birhampur Community Clinic.

^2^LOD = limit of detection (0.15 μg/L).

**Figure 1:**
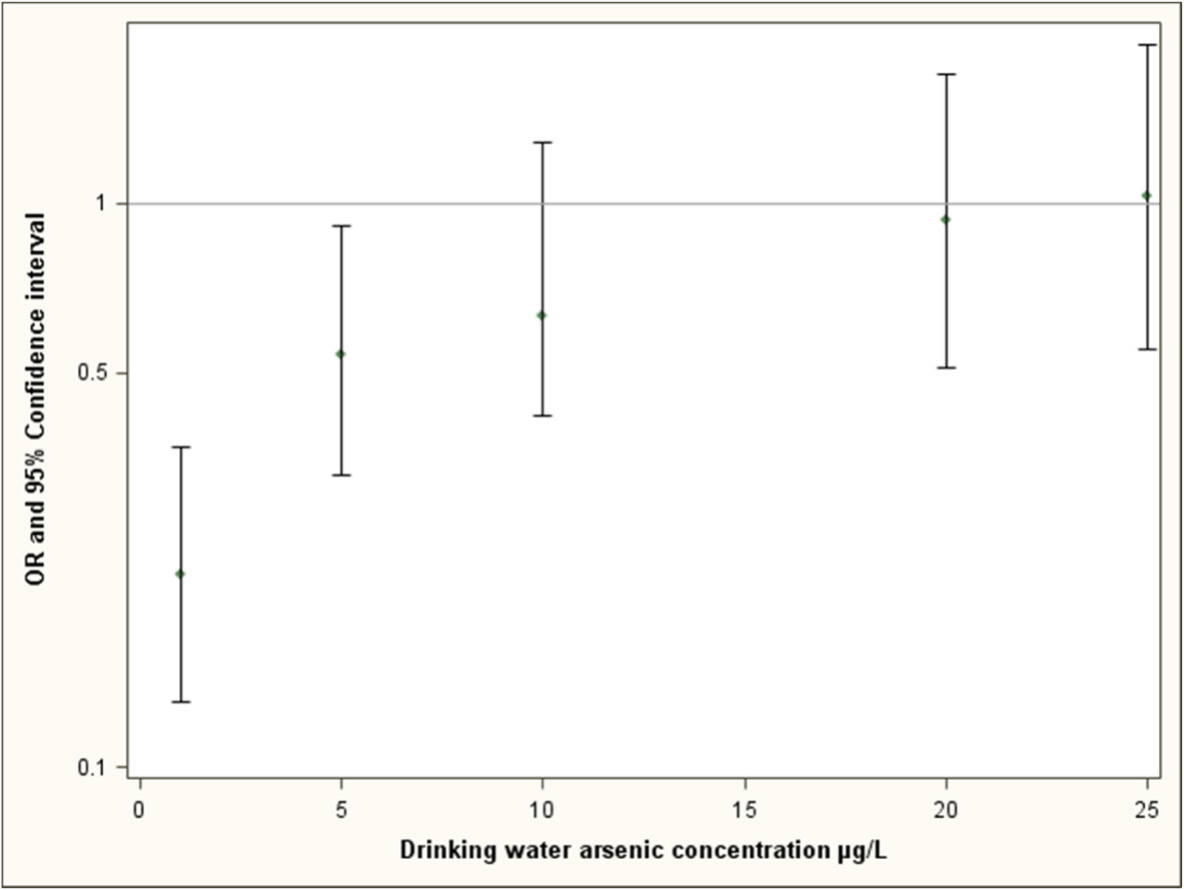
**The predicted odds ratio of periconceptional folic acid use and myelomeningocele from the interaction model at various distributions of inorganic arsenic concentration in drinking water.** Average (mean) values of parental age, child age, child sex and home birth were used to estimate odds ratios.

In adjusted models, drinking water inorganic arsenic concentration collected from wells that were used during the first trimester of pregnancy was not significantly associated with risk of myelomeningocele. In models that tested the interaction between water inorganic arsenic concentrations and periconceptional folic acid use, however, the interaction was statistically significant (Table 3). Using the parameters derived from our logistic regression model, the estimated OR for periconceptional folic acid use demonstrates a decrease in the protective effect of folic acid (OR = 0.22, 95% CI [0.13, 0.37] at 1 μg/L drinking water inorganic arsenic; OR = 1.03, 95% CI [0.55, 1.91]) at 25 μg/L drinking water inorganic arsenic) (Figure 1).

**Table 3:** **Results from weighted logistic regression models with the outcome neural tube defects (n = 112)**

**Variable**	**Main effects model**			**Interaction model**		
	**β**	**OR (95% CI)** ^**1**^	**pValue**	**β**	**OR (95% CI)** ^**1**^	***p*** **Value**
Ln-water As	0.0278	1.03 (0.96, 1.11)	0.45	−0.3190	-	-
Periconceptional folic acid use^2^	−1.2029	0.300 (0.19, 0.47)	<0.0001	1.7280	-	-
Home birth^3^	0.9224	2.51 (1.61, 3.92)	<0.0001	1.0047	2.73 (1.72, 4.32)	<0.0001
Mother’s age in years	0.1649	1.18 (1.10, 1.26)	<0.0001	0.1573	1.17 (1.09, 1.26)	<0.0001
Father’s age in years	−0.0607	0.94 (0.89, 1.00)	0.05	−0.0416	0.96 (0.90, 1.01)	0.17
Child sex^4^	0.9152	2.50 (1.56, 3.99)	0.0001	0.7986	2.22 (1.38, 3.59)	0.001
Child age in months	−0.1305	0.88 (0.84, 0.92)	<0.0001	−0.1337	0.88 (0.94, 0.91)	<0.0001
Ln-water As*prenatal folic acid use				0.4636	-	<0.0001

^1^OR indicates odds of being a CASE.

^2^Reported use = 1, No reported use = 0.

^3^Home birth = 1, Clinic or Hospital birth = 0.

^4^Male = 1, Female = 0.

## Discussion

Our study found a significant interaction between drinking water inorganic arsenic concentration from wells used during the first trimester of pregnancy and reported intake of perinatal folic acid supplements. As drinking water inorganic arsenic concentrations increased from 1 μg/L to 25 μg/L, the estimated protective effect of folic acid use declined (OR = 0.22, 95% CI [0.13, 0.37] to OR = 1.03, 95% CI [0.55, 1.91]). This finding suggests that environmental arsenic exposure reduces the effectiveness of folic acid supplementation in preventing neural tube defects.

Our study is the first in humans to demonstrate that environmental arsenic exposure influences risk of myelomeningocele. At minimum, this study demonstrates that there is a need for surveillance for neural tube defects among populations with high environmental arsenic exposure, and that additional preventive strategies may be required in these areas. On a wider scale, the combination of folate deficiency and arsenic poisoning in Bangladesh may be a setting for an unrecognized epidemic of neural tube defects.

Few studies have investigated the relationship between arsenic exposure during pregnancy and risk of neural tube defects in humans. Most recently, archeological studies in an area of northern Chile characterized by high environmental arsenic levels have shown that the prevalence of spina bifida occulta among mummies was 13.5%, compared with 2.4% in a low-arsenic area believed to have had no other differences in diet or other factors [22]. Investigation of a cluster of anencephalic births near the Texas-Mexico border found that maternal and paternal exposures to arsenic were associated with higher risk of neural tube defects, but this finding was not statistically significant [23]. In Bangladesh, a study of the pregnancy outcomes of 2006 women who regularly consumed drinking water with high arsenic concentration found that there was a small but statistically significant association (OR: 1.005, 95% CI: 1.000-1.010) between arsenic exposure and congenital anomalies, though the study did not specify the nature of these anomalies [24]. During an ecological study in China’s Yangtze River Delta region, the arsenic content of the soil in 40 towns and 80 villages was associated with frequency of congenital anomalies, including neural tube defects [25]. Critiques of the epidemiological literature have noted that human studies of arsenic and neural tube defects have been limited by lack of individual measures of exposure and by lack of measures of exposure during the critical period of early gestation [26].

The strengths of our study include the use of individual measures of exposure, including measurements of the drinking water from wells that mothers identified as their source of water at the time they became aware of their pregnancy—a time point that is very close to the crucial time point in neural tube defect pathogenesis. Our interviews with community members indicated a frequent practice for women to return to their parents’ homes in the months around childbirth, therefore we were careful to identify the exact well used during the first trimester. Though the drinking water was collected from the identified wells approximately one year after the onset of pregnancy, arsenic concentrations have been shown to be stable over a one year time period, with the most recent study in Araihazar, Bangladesh, demonstrating variability of 10% to 30% in well samples collected every 2 to 4 weeks over a period of 3 years [27]. In our study, drinking water inorganic arsenic concentration was used as a proxy for *in utero* arsenic concentration, and drinking water arsenic concnetration may not accurately reflect the internal arsenic dose encountered by the developing embyro. Previous studies in humans support placental transfer of arsenic; studies of pregnant women in Argentina and Bangladesh have shown that the arsenic concentration in cord blood is similar to that in maternal blood [28,29].

Another reason our study may have found associations is that the arsenic concentrations in our study were much higher than those observed in other studies. For example, among controls in the 2006 cluster case–control study conducted along the Texas/Mexico border, less than 2% of women were found to have inorganic arsenic concentrations greater than 10 μg/L in drinking water [23], whereas in this study the drinking water inorganic arsenic concentration was greater than 10 μg/L in 55.5% of controls, and the maximum drinking water arsenic concentration (506 μg/L) was over 50 times that threshold.

An additional strength of our study is that we minimized the potential recall bias by validating folic acid use reports with biomarkers. This comparison has considerable limitations, as we were only able to measure plasma folate after pregnancy. Ours is the first study in Bangladesh to measure plasma folate concentrations among mothers of children with neural tube defects, and we can infer from the strong correlation between the two measures that women who reported periconceptional folic acid use have more access to folate containing foods and/or folic acid supplements. Nevertheless, the potential for recall bias is inherent in all retrospective studies and is an important limitation of this study.

Folate is the centerpiece of the one-carbon metabolism pathway that has a key role in reactions that are important in many key biological processes including synthesis of DNA, and for the generation of methyl groups used in a multitude of important reactions. Arsenic is metabolized in humans through sequential addition of methyl groups acquired from the one-carbon metabolism pathway. In trials, folic acid supplementation has been shown to reduce total blood arsenic concentrations by increasing the methylation of inorganic arsenic into less toxic species [30]. One possible explanation for the observed interaction between arsenic and folic acid supplementation is that arsenic depletes the supply of methyl donors necessary for reactions important in normal neural tube development.

Arsenic may also increase the risk for neural tube defects by disruption of maternal glucose homeostasis. Two well-established risk factors for neural tube defects are maternal gestational diabetes and prepregnancy obesity[31-36]. Accumulating evidence has shown an increase risk of type 2 diabetes in general populations exposed to arsenic [37-39], and there is growing evidence that pregnant women also experience increase risk of dysregulated glucose homeostasis with arsenic exposure [40]. Although the mechanisms underlying the risks of gestational diabetes remain unclear, there is evidence from experimental models that *in utero* hyperglycemia can cause excess generation of reactive oxygen species due to mitochondrial dysfunction [41] and activation of programmed cell death (apoptotic) signaling cascades [42]. None of the women in our study reported a diagnosis of gestational diabetes, but subclinical alterations in glucose regulation due to arsenic toxicity are likely to be present, and may play a role in modulating risk of neural tube defects.

Though the interaction between water arsenic concentration and reported folic acid use was highly significant, our study did not demonstrate a significant primary (or main) effect of drinking water arsenic concentrations on myelomeningocele risk. This is most likely because our pilot study was underpowered, though it is also possible that even higher arsenic exposures are needed to demonstrate a primary effect. Another potential explanation is that our study was designed to identify myelomeningocele at the time of birth or presentation to medical care; a surveillance system following all pregnancies might have identified a more severe phenotype. Infants with arsenic-induced neural tube defects may have died during gestation, or were otherwise undiscovered. Another possible explanation for why we did not find a main effect of arsenic exposure comes from the multifactorial threshold model of neural tube defects that posits that neural tube defects result from an additive contribution of several risk factors, which are each individually insufficient to disrupt neural tube closure [43]. Despite our attempts to adjust for confounding and selection bias, residual differences between the study groups and study sites are still possible, and thus, more detailed collection of important covariates including socioeconomic status, nutritional intake and folate status is needed in future studies.

Our findings raise important questions about the risk of neural tube defects in arsenic-endemic areas and the effectiveness of folic acid supplements. Larger, prospective studies with more refined measures of folic acid supplementation and folate status of mothers are needed.

## Conclusions

Environmental arsenic exposure may reduce the protective effects of folic acid supplementation in high exposure areas, such as Bangladesh. More surveillance for neural tube defects is needed in arsenic endemic areas. Additional preventive measures, such as higher doses of folic acid, as well as early detection and management of maternal diabetes, may be needed in areas with high levels of environmental arsenic exposure.

## Abbreviations

DCH: Dhaka community hospital
LOD: Limit of detection
IPW: Inverse probability weighting
BCC: Birhampur community clinic
SCC: Sirajdikhan community clinic
